# Disease Modifiers of Inherited *SCN5A* Channelopathy

**DOI:** 10.3389/fcvm.2018.00137

**Published:** 2018-10-01

**Authors:** Arie O. Verkerk, Ahmad S. Amin, Carol Ann Remme

**Affiliations:** ^1^Department of Clinical and Experimental Cardiology, Heart Centre, Academic Medical Center, Amsterdam, Netherlands; ^2^Department of Medical Biology, Academic Medical Center, Amsterdam, Netherlands

**Keywords:** Na_V_1.5, LQT3, Brugada syndrome, conduction, co-morbidities

## Abstract

To date, a large number of mutations in *SCN5A*, the gene encoding the pore-forming α-subunit of the primary cardiac Na^+^ channel (Na_V_1.5), have been found in patients presenting with a wide range of ECG abnormalities and cardiac syndromes. Although these mutations all affect the same Na_V_1.5 channel, the associated cardiac syndromes each display distinct phenotypical and biophysical characteristics. Variable disease expressivity has also been reported, where one particular mutation in *SCN5A* may lead to either one particular symptom, a range of various clinical signs, or no symptoms at all, even within one single family. Additionally, disease severity may vary considerably between patients carrying the same mutation. The exact reasons are unknown, but evidence is increasing that various cardiac and non-cardiac conditions can influence the expressivity and severity of inherited *SCN5A* channelopathies. In this review, we provide a summary of identified disease entities caused by *SCN5A* mutations, and give an overview of co-morbidities and other (non)-genetic factors which may modify *SCN5A* channelopathies. A comprehensive knowledge of these modulatory factors is not only essential for a complete understanding of the diverse clinical phenotypes associated with *SCN5A* mutations, but also for successful development of effective risk stratification and (alternative) treatment paradigms.

## Introduction

To date, an increasing number of mutations in *SCN5A*, the gene encoding the pore-forming α-subunit of the primary cardiac Na^+^ channel (Na_V_1.5), is found in patients with a wide range of electrocardiogram (ECG) abnormalities and cardiac syndromes (1–3). Although they are all due to mutations in the same ion channel, these syndromes show a myriad of phenotypes (4). While this may be partly explained by mutation-specific biophysical changes in the current generated by Na_V_1.5 channels (here named Na^+^ current, I_Na_), it has now become clear that a single mutation in *SCN5A* may also result in a large number of disease phenotypes within one and the same family [for review, see (2)]. Also, disease severity often varies significantly among affected individuals, with some *SCN5A* mutation-positive patients suffering from life-threatening arrhythmias at young age while others do not display any clinical signs (i.e., reduced and incomplete penetrance).

At this moment, clinical management of *SCN5A* mutation-positive patients is hindered by this reduced penetrance as well as by the considerable variation in disease severity and risk of sudden cardiac death (SCD) observed in affected individuals. Cardiac and non-cardiac modulatory factors and co-morbidities are supposed to modify disease severity and expressivity, however, till now they are largely unexplored. A major reason for the lack of detailed information on such disease modifiers in *SCN5A-*mutation related disorders is the large genetic heterogeneity between individual patients. In addition, different mutations result in different biophysical alterations and thus give rise to further variability between individuals. In this review, we provide an updated summary of presently identified cardiac disease entities secondary to *SCN5A* mutations, and give an overview of a broad spectrum of concomitant disorders and conditions which may modify disease severity and expressivity of *SCN5A* channelopathies.

## Cardiac disorders associated with *SCN5A* mutations

Na_V_1.5 channels are widely distributed in the mammalian heart, but the number of channels (5–7) and their electrophysiological function (6, 8–10) may differ between various parts of the heart. Consequently, *SCN5A* mutations can lead to multiple cardiac disease phenotypes, and even considerable overlap may exist, named “overlap syndrome,” between these cardiac clinical entities (2). Aside from the heart, Na_V_1.5 channels are also expressed in other tissues throughout the body, and *SCN5A* mutations therefore are also associated with extracardiac phenotypes, including gastrointestinal dysfunction (11) and epilepsy (12). Below, we first provide a brief overview of the Na_V_1.5 channel and I_Na_ properties and subsequently introduce briefly the various *SCN5A*-related cardiac disorders in relation to the associated biophysical Na_V_1.5 channel defects.

### Na_V_1.5 structure and function

**Figure 1:**
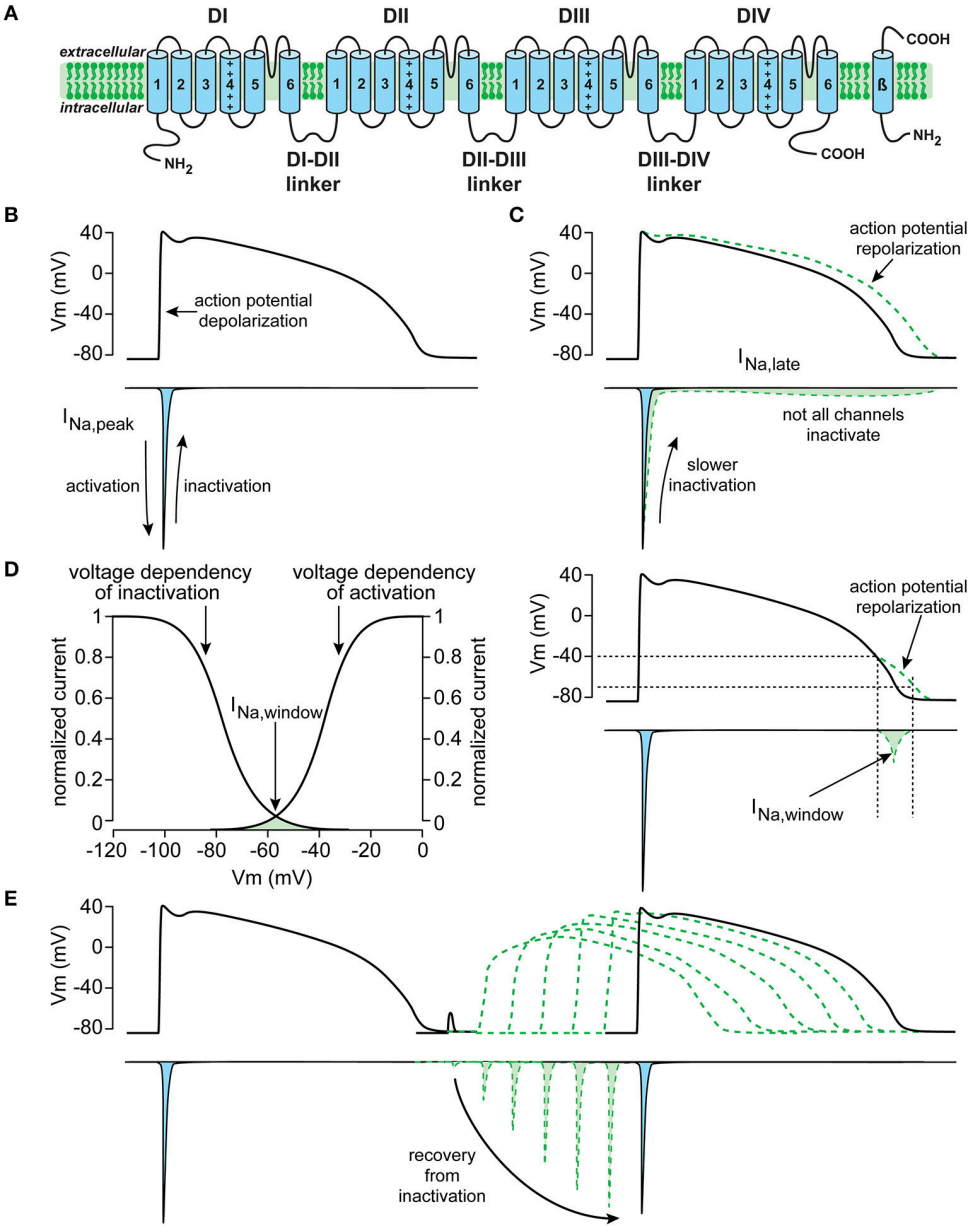
Schematic drawings of the cardiac sodium channel Na_V_1.5 encoded by the *SCN5A* gene **(A)**, and important biophysiological properties and function of the current generated by *SCN5A* (here named I_Na_), including peak I_Na_ **(B)**, late I_Na_ **(C)**, window I_Na_ **(D)**, and recovery from inactivation **(E)**.

As reviewed in detail elsewhere (13), the Na_V_1.5 protein is formed by four homologous domains (D1–DIV) each composed of six transmembrane spanning helices (S1–S6) (Figure 1). Na_V_1.5-based channels are voltage dependent and open upon depolarization, resulting in a rapid activation of I_Na_. In working myocytes, this I_Na_ is large and generates the fast action potential (AP) depolarization (6, 14). Typically, Na_V_1.5 channels also close rapidly due to inactivation. This fast inactivation, together with the reduction in driving force of Na^+^ ions occurring during the AP upstroke, results in a rapid decrease of I_Na_ (Figure 1B). Although most Na_V_1.5 channels show fast inactivation, some channels may inactivate slower and/or incompletely. Consequently, a small persistent or late I_Na_ current is generated (Figure 1C), which may affect AP repolarization (15). Moreover, a small overlap exists between the voltage dependence of activation and inactivation. Therefore, Na_V_1.5 channels can activate but are not inactivated completely, resulting in a small I_Na_ at this range of membrane potentials, named the “window current” (Figure 1D). Such a window current also contributes to the AP repolarization phase. In addition, late and/or window I_Na_ may also affect pacemaker activity of sinoatrial nodal (SAN) cells (8, 10) and excitability (16). Upon return to hyperpolarized potentials, i.e., during or following the AP repolarization, Na_V_1.5 channels can quickly recover from inactivation (14). The speed of recovery from inactivation regulates Na_V_1.5 channel availability for subsequent APs, and is therefore responsible for the refractory period (17).

### *SCN5A*-related disorders

Brugada syndrome (BrS) is characterized on the ECG by ST-segment elevation in the right-precordial leads V1 to V3. BrS is associated with ventricular arrhythmias and SCD, which occur particularly during rest and sleep in apparently healthy and young (age < 40 years) individuals (18). The characteristic ST-segment elevation of the ECG is often variably present, and can be unmasked by I_Na_ blockade or exercise [see (18)]. *SCN5A* mutations linked to BrS are so called “loss-of-function” mutations, which typically result in a decreased I_Na_ (1). This reduction in I_Na_ may be due to decreased trafficking and membrane channel expression and/or altered gating properties of the channel resulting in disruption of voltage dependency of (in)activation, accelerated speed of inactivation, or slowed recovery from inactivation.

Long QT syndrome (LQTS) is characterized by a QT-interval prolongation on the ECG accompanied by an enhanced risk for SCD as a result of ventricular tachyarrhythmias. LQTS type 3 (LQT3), the subtype caused by *SCN5A* mutations, is associated with bradycardia and arrhythmias and/or SCD occurring mostly at slow heart rates such as during rest or sleep (19). *SCN5A* mutations underlying LQT3 are typically “gain-of-function” mutations inducing various biophysical alterations (such as slower I_Na_ inactivation, larger late I_Na_, larger window I_Na_, and/or increased I_Na_ density (1), all leading to an enhanced I_Na_ function during the AP repolarization phase and consequent AP prolongation.

Atrial fibrillation (AF), a rapid and irregular beating of the atria, is mostly found in elderly patients with structural alterations in the heart. Evidence is increasing that AF in young patients with structurally normal hearts may also be hereditary. In familial forms of AF, both *SCN5A* loss-of-function and gain-of-function mutations have been identified (20). The gain-of-function can be due to various gating changes including negative shifts in voltage dependence of activation, positive shifts in voltage dependence of inactivation, slower current inactivation, and faster recovery from inactivation [see (16), and primary references cited therein]. Loss-of-function can be the consequence of reduced I_Na_ density (21) or of a negative shift in voltage dependence of inactivation (22).

Sick sinus syndrome (SSS) is described as the “intrinsic inadequacy of the SAN to perform its pacemaking function due to a disorder of automaticity and/or inability to transmit its impulse to the rest of the atrium” [see (23)]. A number of *SCN5A* mutations have been associated with inherited SSS, and interestingly these can be both loss-of-function and gain-of-function mutations. Consequently, the occurrence of SSS has a considerable overlap with BrS (24) and LQT3 (25, 26). Loss-of-function, i.e., a reduction of I_Na_ availability, decreases the speed of the diastolic depolarization phase of SAN cells and thereby pacemaker activity (25). The overlap of SSS and gain-of-function mutations associated with LQT3 is more complex. Although an increase in late I_Na_ results in faster pacemaker activity (25), the concurrent changes in I_Na_ density and the shifts in voltage dependency of activation and inactivation counteract the enhanced late I_Na_, resulting in a slower pacemaker activity (25).

Progressive cardiac conduction defect (PCCD) is characterized by progressive conduction slowing through the His-Purkinje system. PCCD is associated with PQ- and QRS-interval prolongation, complete atrio-ventricular (AV) and right and/or left bundle branch block, syncope and SCD. PCCD is often observed in BrS patients, and similar to BrS, is due to loss-of-function mutations (18).

Multifocal ectopic Purkinje-related premature contraction (MEPPC) is characterized by frequent premature ventricular contractions originating from the Purkinje system, especially at rest (16). The *SCN5A* mutations underlying MEPPC are typically gain-of-function mutations due to an increased window I_Na_, faster recovery from inactivation and/or increased channel availability of Na_V_1.5 (see (16), and primary references cited therein).

Sudden infant death syndrome (SIDS) is characterized by the sudden unexplained death of a seemingly healthy infant younger than 1 year. SIDS is a disease with multiple pathophysiological mechanisms (27), and cardiac ion channel gene mutations appear to be involved in approximately 20% of the cases of SIDS, from which more than half of the mutations are related to I_Na_ [for review, see (28)]. These may include mutations in *SCN5A*, but also in the I_Na_-modulatory β-subunits (*SCN3B* and *SCN4B*) and other “regulatory genes” (*CAV3, SNTA1*, and *GPD1-L*), which could result in either I_Na_ loss-of function or gain-of-function mutations [see (28, 29), and primary references cited therein].

Dilated cardiomyopathy (DCM) is a structural heart disease characterized by dilated chambers, pump failure, and arrhythmia. DCM is a multifactorial disorder with several proposed pathophysiological mechanisms (30), including *SCN5A* mutations (31). Both loss-of-function and gain-of-function are associated with DCM, but the pathophysiological mechanisms of DCM secondary to *SCN5A* mutations are not exactly known (2, 32). As reviewed by Wilde and co-workers, DCM may be: (i) secondary to *SCN5A* mutation induced arrhythmias and/or bradycardia; (ii) due to increased late I_Na_ and consequent changes in intracellular Na^+^ and Ca^2+^; or (iii) secondary to the non-electrical role of Na_V_1.5 as a potential anchoring protein for structural and cytoskeletal proteins (33).

Arrhythmogenic right ventricular cardiomyopathy (ARVC) is an inherited cardiomyopathy characterized by fibrofatty replacement of the right ventricle, ventricular arrhythmias, and SCD (34). Up to 60–70% of the ARVC index cases carry a causal desmosomal [such as plakophilin-2 (*PKP2*) or desmoglein-2 (*DSG2*)] gene mutation, but various non-desmosomal genes may also be involved (35, 36), including *SCN5A* (37). Although the percentage of pathogenic *SCN5A* mutations in ARVC is very low, *PKP2* knockdown and overexpression of *Dsg2* mutations both result in a decrease in I_Na_ (38, 39), and such a decrease in I_Na_ is proposed to be a critical factor in arrhythmogenesis in ARVC (40).

## Variable expressivity in *SCN5A* channelopathy

**Figure 2:**
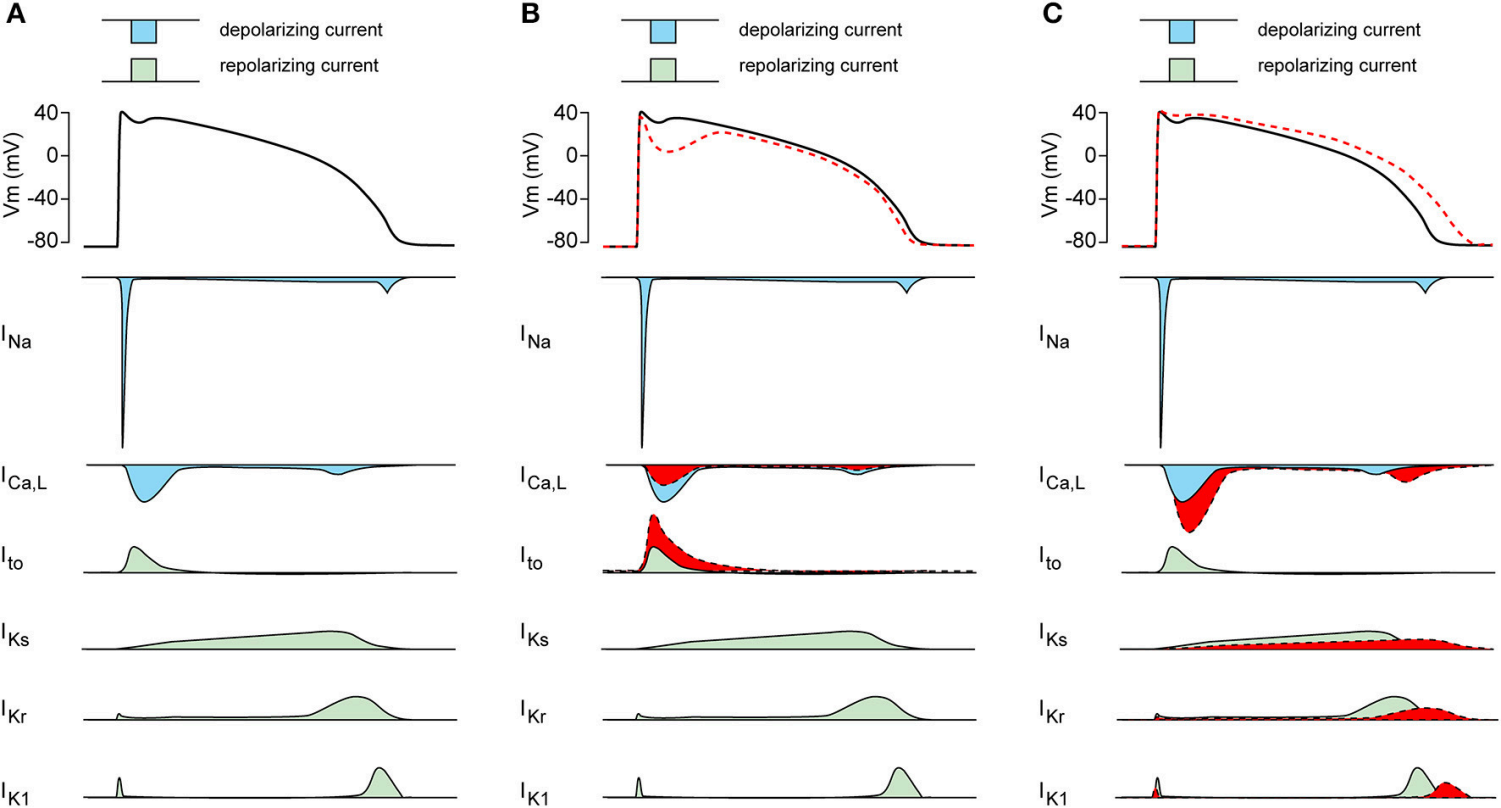
**(A)** Schematic drawing of the cardiac action potential (AP) and its underlying membrane currents. I_Na_, Na^+^ current; I_Ca,L_, L-type Ca^2+^ current; I_to_, transient outward K^+^ current; I_Ks_, slow component of the delayed rectifier K^+^ current; I_Kr_, rapid component of the delayed rectifier K^+^ current; I_K1_, inward rectifier K^+^ current. **(B)** Schematic drawing of AP plateau suppressing ion channels changes (in red). **(C)** Schematic drawing of AP prolonging ion channels changes (in red).

Patients harboring *SCN5A* mutations demonstrate a significant variability in disease expression (41). Obviously, such variability in *SCN5A*-releated diseases can be due to different severities of the I_Na_ biophysical defect, with truncating *SCN5A* loss-of-function mutations resulting in more pronounced conduction slowing than missense *SCN5A* mutations (42). The range of biophysical alterations induced by a particular genetic defect in *SCN5A* (1) may also determine the capability of that mutation to cause cardiac rhythm disorders. Importantly, the variability in *SCN5A*-releated disease severity and expressivity is also present in family members carrying the same mutation, as exemplified in a large Dutch family with the *SCN5A*-1795insD “overlap syndrome” mutation (43). Some mutation carriers in this family display predominantly loss-of-function phenotypes with BrS and/or conduction disease, while other family members carrying this mutation show mainly a gain-of-function phenotype resulting in QT-prolongation (44). In addition, and apart from family members with a clear phenotype, other family members carrying the same *SCN5A*-1795insD mutation appear unaffected (43). Thus, independent of the mutation-specific effects, individual-specific factors also appear to contribute importantly to the regulation of disease expressivity and severity in *SCN5A* channelopathy. Moreover, the variability in Na_V_1.5 disease expression and severity is not only related to I_Na_ defects, but likely also closely related to other cardiac ion channels which contribute to the cardiac AP. Apart from I_Na_, the AP morphology is the consequence of a fine balance between the inwardly directed L-type Ca^2+^ current (I_Ca,L_; Ca_V_), and various outwardly directed K^+^ currents (K_V_) including the transient outward K^+^ current (I_to_), the inward rectifier K^+^ current (I_K1_) and the slow and rapid delayed rectifier K^+^ currents (I_Ks_ and I_Kr_, respectively) (Figure 2A). Changes in these Ca_V_ and various K_V_ currents may affect the expressivity of *SCN5A* channelopathies. For example, a decrease in I_Ca,L_ and/or increase I_to_ (Figure 2B, in red) may increase phase-1 repolarization and lower the AP plateau phase which may promote ST-segment elevation and BrS (45), while an increase in I_Ca,L_ and/or decrease of K_V_ currents (Figure 2C, in red) will result in longer APs thus promoting LQT3 (46).

**Figure 3:**
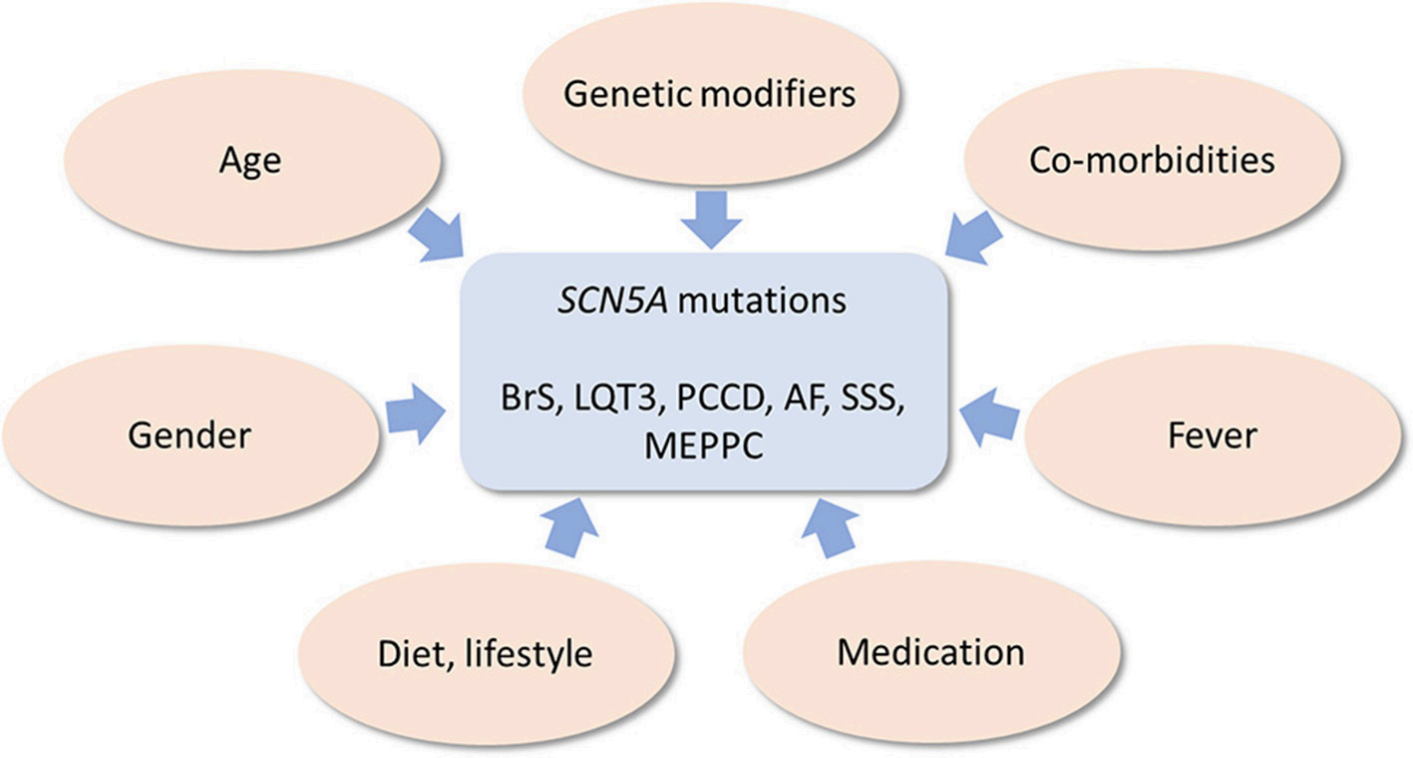
Schematic drawing of genetic and non-genetic disease modifiers of inherited cardiac *SCN5A* channelopathies.

Below, we provide an overview of various known genetic and non-genetic disease modifiers of inherited cardiac *SCN5A* channelopathies, which are summarized in Figure 3.

### Genetic modifiers of *SCN5A* channelopathy

Genetic background and modifiers are considered important determinants of disease expressivity and/or severity in *SCN5A* channelopathies, especially among patients carrying the same mutation (47–49). This has been clearly demonstrated in experimental studies where the impact on genetic variability on disease severity was evaluated in two distinct strains (129P2 and FVBN/J background) of mice carrying the *Scn5a*-1798insD/+ mutation, the equivalent to *SCN5A*-1795insD in humans. A more severe phenotype was present in 129P2 mice as compared to FVBN/J mice (50, 51). In addition, subsequently identified potential modifiers of conduction disease severity were found. Comparison of cardiac gene expression between the 129P2 mice and FVBN/J mice demonstrated that *Scn4b* (encoding a ß-subunit of sodium channels) is an important modifier of conduction disease severity (52). Furthermore, by performing a system genetics approach on F2 progeny arising from these two mouse strains, we showed that *Tnni3k* (encoding troponin 1 interacting kinase) is another modulator of AV conduction (53). These genetic studies clearly underline the relevance of genetic background and genetic modifiers in sodium channelopathy.

Single nucleotide polymorphisms, frequently observed in the general population, may further determine disease expressivity and/or severity. For example, H558R is the most commonly found *SCN5A* polymorphism (with a 9–36% prevalence), and its distribution varies between different ethnic populations (54). Co-existence of this polymorphism and *SCN5A* mutations may affect the functional consequences of the latter, including plasma-membrane targeting of Na_V_1.5, I_Na_ density and/or I_Na_ gating properties (55–60). Moreover, a combination of specific polymorphisms [haplotype (HapB)] within the *SCN5A* promoter region may affect conduction in BrS patients (61). HapB is frequently present in Asians, and may therefore partly explain the high prevalence of BrS in individual with an Asian background. In addition, polymorphisms in non-*SCN5A* genes may also contribute to disease expressivity in sodium channelopathy. For example, Groenewegen et al. (62) demonstrated that phenotype severity of *SCN5A*-D1275N mutation carriers was importantly modulated by 2 closely linked polymorphisms forming a haplotype within the promotor region of the *GJA5*, the gene underlying the atrial-specific connexin-40 gap junction protein. *SCN5A*-D1275N mutation carriers homozygous for the *GJA5* promoter polymorphisms exhibited atrial standstill, while carriers without or with only a heterozygous *GJA5* promoter polymorphism displayed only a mild PR-interval prolongation (62).

Additionally, genetic variation due to the presence and relative expression of two important *SCN5A* alternatively spliced variants, i.e., *SCN5A*-Q1077del and *SCN5A*-Q1077 (63), may further modulate sodium channelopathy severity. The BrS phenotype severity associated with the *SCN5A*-G1406R mutation was enhanced in combination with the Q1077 variant (64). Q1077del has furthermore been shown to modulate I_Na_ density, gating properties, and recovery from inactivation of *SCN5A* mutations associated with DCM (65).

### Non-genetic modifiers of *SCN5A* channelopathy

#### Gender

Gender is a clear modifier of disease severity in *SCN5A* channelopathy, exemplified by the preponderance of BrS in males (66), and LQT3 in females especially in the 30–40 year age range (67). In addition, within one family with the G1406R loss-of-function mutation, females were found to have mostly cardiac conduction defects whereas males showed predominantly a BrS phenotype (47). Gender, and particularly sex hormones, has a significant impact on ion channels responsible for repolarization, and is associated with a larger I_Ca,L_ and smaller I_to_ and consequently higher QTc values in females [see (68), and primary references cited therein]. This lower repolarization reserve intrinsic to female hearts is thought to augment the detrimental impact of a mutation-induced late I_Na_. Barajas-Martinez and colleagues reported a higher I_Na_ magnitude in male epi- and endocardial myocytes compared to female (69). In addition, they found in females a larger ventricular transmural dispersion of I_Na_ density. They suggested that in the setting of decreased I_Na_, epicardial myocytes display more easily all-or-none repolarization leading to BrS in males (with a smaller I_Ca,L_ and larger I_to_), while females with a smaller I_Na_ are more sensitive to loss of conduction velocity.

#### Age

Age is another determinant of severity and expressivity of *SCN5A* channelopathies (70–72). For example, carriers of the *SCN5A*-1795insD mutation show QT-interval prolongation and conduction disorders from birth, while features of BrS mostly develop later in life (72). While peak I_Na_ density and I_Na_ availability (i.e., AP upstroke velocity) does not appear to change with age (73, 74), aging may result in an acceleration of I_Na_ inactivation and an enhanced use-dependent decrease in I_Na_ (73). In addition, aging myocytes also show AP prolongation secondary to both an increase in late I_Na_ and a reduction in K_V_ currents (74). These ion channel changes, together with a prolonged AP (74) (hence, a shorter time for recovery from inactivation) may promote BrS, conduction delay, and LQT3. Furthermore, fibrosis due to aging is thought to play another major role in modulating conduction and repolarization disorder severity (75–77).

#### Medication

It is well known that many clinically used antiarrhythmic, psychotropic, and anesthetics drugs may induce type-1 ECG and/or arrhythmias in BrS patients (78, 79). These drugs with potential adverse effects for BrS patients (for overview, see the website www.brugadadrugs.org) are known to block I_Na_ and/or Ca_V_ currents significantly, thereby increasing the susceptibility for BrS. In addition, many clinically used drugs are also known to result in QT-interval prolongation (71, 80). For an overview of QT-interval prolonging drugs, see the website www.QTdrugs.org. These drugs prolong the QT-interval due to blockade of I_Kr_ or I_Ks_, rather than an increase of late I_Na_, and increase the arrhythmia risk in patients with inherited LQTS, including LQT3 (81).

#### Lifestyle

Evidence is increasing that lifestyle can have a significant impact on *SCN5A* channelopathies by either a direct modulation of I_Na_ properties or indirectly via impacting on K_V_ and Ca_V_ channels, making the heart more sensitive to (the consequences of) *SCN5A* mutations.

##### Alcohol

Alcohol consumption has been associated with BrS (82). Alcohol intoxication may have pro-arrhythmic actions through I_Na_ channel inhibition, thereby mimicking the actions of I_Na_ blocking drugs (83, 84). Furthermore, ethanol shortens the AP through multiple effects on Ca_V_ and K_V_ channels [see (84), and primary references cited therein]; hence, alcohol could theoretically reduce QT-interval prolongation and arrhythmias in the setting of LQT3. On the other hand, episodic excessive alcohol intake is associated with an increase in QT duration dispersion due to cardiac autonomic imbalance (85), which may in fact promote repolarization abnormalities.

##### Recreational drug use

Recreational drug use is another well-known factor in BrS, especially cocaine (79). Cocaine has multiple indirect and direct effects on the electrical activity of the heart as demonstrated by increases in PR-, QRS-, and QT-intervals due to inhibition of Ca_V_, K_V_ and Na_V_ currents (86). The decrease in I_Na_ appears to be caused by slower recovery from inactivation in combination with a shift in voltage dependency of inactivation (86). The cocaine-induced QT-prolongation is importantly due to a blockade of I_Kr_, and predisposes to the occurrence of EADs and TdP (86).

##### Tobacco

Tobacco use has many detrimental effects on general health. In addition, nicotine and carbon monoxide (CO), a major component of smoke, also cause changes in cardiac development as well as ion channel remodeling (87, 88). For example, a low plasma concentration of nicotine increased peak I_Na_ and late I_Na_, with shifts in both inactivation and activation kinetics resulting also in a larger I_Na_ window current (88). In addition, sublethal CO exposure is frequently associated with cardiac arrhythmias, and it has been demonstrated that its effects may be due to Na_V_1.5 channel modulation, causing an increase in late I_Na_, but a decrease of peak I_Na_ (89).

##### Exercise

Exercise, especially swimming, may trigger most types of LQTS (90), but paradoxically appears to lower arrhythmia risk in LQT3 patients (91). On the other hand, exercise may aggravate the ECG defects observed in BrS patients (92). These acute effects of exercise on BrS and LQT3 may be explained by vagal activity and rapid heart rates, resulting in less recovery from inactivation in combination of a lower driving force of Na^+^ ions due to intracellular Na^+^ accumulation (91–93). Regular low intensity exercise and endurance training can also lead to structural and electrical remodeling of the heart [for review, see (92)]. A well-known effect of exercise training is a reduction of resting heart rate, partially via a decrease of the hyperpolarization-activated current, I_f_ (94). Theoretically, such a lower resting heart rate may itself increase the susceptibility to both BrS and LQT3. On the other hand, exercise training does not affect the expression of *SCN5A* mRNA (95), but reduces I_to_ in epicardial myocytes thereby reducing the transmural gradient of I_to_ significantly (96). This could potentially suppress BrS, but may increase LQT3 due to AP prolongation (96).

##### Diet and dietary supplements

Diet may have both beneficial and detrimental effects on *SCN5A*-related diseases, but underlying mechanisms appear complex. For example, acute application of polyunsaturated fatty acids, in particular those of the n-3 class (PUFAs), inhibits I_Na_ (97) and therefore may facilitate BrS. Yagi et al. (98), however, suggested that n-3 PUFAs may prevent ventricular fibrillation in BrS, likely due to additional blockade of various other cardiac ion channels (68), including I_to_ (99). High cholesterol and fat intake may constitute additional diet-related modulatory factors. Both are associated with a slower recovery from I_Na_ inactivation, but with a more negative voltage dependence of I_Na_ activation, which may lower the threshold for excitation of Na_V_1.5 channels (100). To date, the clinical impact of high cholesterol and fat intake on LQT3 and BrS patients are as yet unknown. Interestingly, consuming a large meal, resulting in vagal stimulation, may trigger sudden cardiac arrest in BrS (101, 102). In addition, glucose load (alone and in combination with insulin infusion), as well as Thai high glycemic index (HGI) meals are known to affect ST-segment elevation in BrS patients (see (103), and primary references cited therein). The mechanism behind this effect may be related to glucose-induced insulin secretion. In myocytes, insulin results in activation of the Na/K pump (104), and consequently, in an increased outwardly directed current during the AP thereby theoretically promoting repolarization. On the other hand, insulin in myocytes enhanced the depolarizing I_Ca,L_ (105), while it inhibits I_Kr_ (106) and I_Ks_ (107), thereby prolonging the QTc in humans (108) which may favor LQTS. More studies are required to elucidate the exact role of glucose/insulin on BrS and LQT3, and to explain the so-called diabetic death-in-bed syndrome as mentioned by Skinner et al. (109). Furthermore, high salt and glucose intake can result in hypertension and diabetes, respectively. Both diseases have significant impact on ion channel function, and hence likely also modulate disease expressivity and severity in the setting of *SCN5A* mutations (see also below).

These days, dietary supplements, natural drugs, and/or traditional Chinese medicines are increasingly used (110). Some ingredients in these preparations shorten the cardiac AP due to I_Na_ and I_Ca,L_ inhibition [for review, see (110)], thus caution for BrS patients seems appropriate. Other compounds, such as Wenxin Granule [for review, see (111)], may however have a therapeutic effect on BrS. Although Wenxin Granule was shown to reduce I_Na_, it also suppressed the electrocardiographic and arrhythmic manifestations of BrS due to inhibition of I_to_ (112). It has also been shown to reduce late I_Na_ (113, 114), and therefore may also have an impact in LQT3 patients. Resveratrol, a polyphenol compound that is primarily derived from grapes, also inhibits late I_Na_ as well as I_Ca,L_ (110); hence, LQT3 patients may have some benefit from such natural and readily available supplements. Another example of a traditional Chinese medicine is dimethyl lithospermate B (dmLSB), an extract of Chinese herbal Danshen. dmLSB slows I_Na_ inactivation, thereby potentially eliminating the arrhythmogenic substrate responsible for BrS (115). Other ingredients of natural drugs and/or traditional Chinese medicines are known to prolong the AP due to K_V_ blockade which may consequently predispose to arrhythmias in LQT3 patients [for review, see (110)]. Finally, apart from direct action om membrane currents, diet and dietary supplements may lead to electrolyte changes, which may have an indirect impact on ion channel function and thereby modify disease expression. For example, higher K^+^ levels may shorten the QT-interval in LQT3 patients while hypokalemia is a well-known trigger of QT-interval prolongation and arrhythmias in patients with LQTS (116). Thus, diet and dietary supplements may impact on various *SCN5A*- related conditions, but randomized clinical trials are required to assess their potential beneficial and/or detrimental effects in *SCN5A* channelopathy patients.

##### Environmental conditions

Environmental conditions should also be considered as potential disease modifiers in *SCN5A* channelopathies. Particulate air pollution, for example, has been associated with increased QTc duration (117), and thus may theoretically increase disease severity in LQT3. In addition, sudden noises are well-known to trigger *SCN5A*-related arrhythmias (1), but evidence is increasing that more chronic, environmental noise pollution also increased incidence of arrhythmias, especially AF (118). The exact mechanism is yet unknown, but noise is a non-specific stressor that activates the autonomous nervous system and endocrine signaling with multiple effects on human health [for review, see (119)].

#### Fever

Some *SCN5A* mutations may induce BrS-associated symptoms especially during fever episodes, with may be due to changes in I_Na_ channel gating properties in response to increasing temperature (120, 121). We and others have shown that specific *SCN5A* mutations promote slow inactivation of I_Na_ at higher temperatures (i.e., enhanced slow inactivation), thereby causing reduced peak I_Na_ availability (122, 123). To date, specific LQT3-associated *SCN5A* mutations which display enhanced temperature sensitivities have not been described (121). In general, increased temperature does not affect the ratio between late and peak I_Na_ (124), but enhances the transmural repolarization dispersion thus facilitating the occurrence of torsade de pointes (TdP) during LQTS (125). While these observations suggest an increased sensitivity for LQT3 during fever, evidence for this is as yet lacking.

#### Diabetes

Patients with diabetes are more vulnerable for the development of arrhythmias, independent of other risk factors like hypertension and atherosclerosis (126). QT-interval prolongation is more often observed in diabetic patients as compared to non-diabetic individuals (127). QT prolongation, due to downregulation of K_V_4 channels, is also observed in rat and mouse models of diabetes (126, 128). Interestingly, diabetic mice also show an enhanced late I_Na_ (126). It is therefore plausible that diabetes increases disease severity in LQT3 patients, but evidence for such a modulatory effect is currently lacking. On the other hand, a decrease in Na_V_1.5 expression and I_Na_ has been reported in rabbit and rat models of diabetes (129, 130), which may have important implications for BrS.

#### Obesity

Obesity, marked by excessive fat accumulation and weight gain, may result in various chronic disorders such as dyslipidemia, insulin resistance, hypertension, hyperglycemia, and type 2 diabetes (131). Thus, it has multiple similarities with a number of other topics discussed in this review. Therefore, it is not unexpected that obesity can lead to various cardiac electrical disorders including AF, (supra)ventricular arrhythmias (128, 132), and LQTS (133). At this moment, it is not known whether obesity impacts on disease expressivity and/or severity in *SCN5A*-related channelopathies. However, given its QT-prolonging effect through an increase in I_Ca,L_ and a decrease of various K_V_ channels (132), it is conceivable that obesity may exacerbate LQT3-associated features. Direct effects of obesity on peak and late I_Na_ have only been investigated in limited fashion, with contrasting results (for review, see (132), and primary references cited therein). Nevertheless, since the number of obese individuals is steadily rising, further studies are essential to elucidate potential obesity-related ion channel remodeling and consequences for arrhythmogenesis in the setting of ion channelopathies.

#### Hypertension

Hypertension may lead to progressive myocardial remodeling, ultimately resulting in the development of cardiac hypertrophy and associated electrical, homeostatic and structural alterations (134). The latter may act synergistically with biophysical alterations secondary to a *SCN5A* mutation resulting in an enhanced pro-arrhythmogenic substrate (135). Due to its progressive nature, the impact of hypertension-induced pro-arrhythmic remodeling is expected to increase with age. Indeed, we have recently demonstrated that co-existing hypertension increased arrhythmia risk and reduced the efficacy of pacemaker treatment in carriers of the *SCN5A*-1795insD mutation above the age of 40 years. Enhanced late I_Na_, a known consequence of hypertrophy, was shown to be at least partly involved and may constitute a promising therapeutic strategy by additionally preventing intracellular sodium/calcium dysregulation (51, 136, 137). Other studies have shown a similar interaction between hypertension and disease severity and outcome, for example in hypertrophic cardiomyopathy (138). Hence, careful monitoring of hypertension and hypertrophy in addition to aggressive anti-hypertensive treatment should be considered in *SCN5A* mutation carriers.

#### Coronary artery disease

Coronary artery disease may enhance the risk for cardiac events in BrS and LQTS patients. Co-existence of BrS and coronary spasm has been observed in Japanese patients (139–142), but not in European patients (143). The relation to *SCN5A* mutations were not mentioned in these studies, but van Hoorn and colleagues found that the prevalence of coronary artery disease was significantly higher among BrS patients with *SCN5A* mutations than among BrS patients without *SCN5A* mutations (144). Interestingly, Kujime and coworkers reported that coronary artery vasospasm could be a risk factor for cardiac events in patients with BrS (145). Coronary artery disease was reported to augment the risk for LQTS-related cardiac events in LQTS patients over age the age of 40 years (146), but no subdivision into the various types of LQTS was performed. The exact reason for such an augmentation is not known, but may be related to longer QTc intervals in patients with coronary artery disease (147, 148). Alternatively, it may be consequent to alterations in the tissue substrate (e.g., ischemia, scar formation, reduced ejection fraction) which may lower the threshold for afterdepolarizations in LQTS, a critical factor in the initiation of torsade de pointes that is thought to be the arrhythmogenic mechanism in LQTS-related cardiac events [see (146)]. Thus, it appears that coronary artery disease may enhance the risk for cardiac events in both BrS and LQTS patients, but further clinical studies are required to substantiate these observations.

## Conclusions

Genetic modifiers, (common) co-morbidities, environmental influences, and life style factors including diet and exercise may modify disease expressivity and severity, and as such significantly modulate the risk for arrhythmia occurrence and survival in *SCN5A* channelopathy. Importantly, the impact of modulatory factors may differ between distinct mutations, but may also vary with age and gender. Hence, clinical management of patients with *SCN5A* mutations should include careful and continuous assessment of co-existing diseases and other modulatory factors, in addition to rigorous treatment of relevant co-morbidities. Identification of disease modifiers will be an essential step in further research related to *SCN5A* channelopathies and may help to design better risk stratification algorithms and to improve development of novel diagnostic and therapeutic strategies.

## Author contributions

All authors listed have made a substantial, direct and intellectual contribution to the work, and approved it for publication.

### Conflict of interest statement

The authors declare that the research was conducted in the absence of any commercial or financial relationships that could be construed as a potential conflict of interest.
